# β-catenin signaling, the constitutive androstane receptor and their mutual interactions

**DOI:** 10.1007/s00204-020-02935-8

**Published:** 2020-10-24

**Authors:** Albert Braeuning, Petr Pavek

**Affiliations:** 1 Department Food Safety, German Federal Institute for Risk Assessment, Max-Dohrn-Str. 8-10, 10589 Berlin, Germany; 2 Department of Pharmacology and Toxicology, Charles University, Faculty of Pharmacy, Heyrovskeho 1203, Hradec Kralove, 500 05 Prague, Czech Republic

**Keywords:** Wnt signaling, Nuclear receptor, Hepatocyte, Liver tumor promotion, Drug metabolism

## Abstract

Aberrant signaling through β-catenin is an important determinant of tumorigenesis in rodents as well as in humans. In mice, xenobiotic activators of the constitutive androstane receptor (CAR), a chemo-sensing nuclear receptor, promote liver tumor growth by means of a non-genotoxic mechanism and, under certain conditions, select for hepatocellular tumors which contain activated β-catenin. In normal hepatocytes, interactions of β-catenin and CAR have been demonstrated with respect to the induction of proliferation and drug metabolism-related gene expression. The molecular details of these interactions are still not well understood. Recently it has been hypothesized that CAR might activate β-catenin signaling, thus providing a possible explanation for some of the observed phenomena. Nonetheless, many aspects of the molecular interplay of the two regulators have still not been elucidated. This review briefly summarizes our current knowledge about the interplay of CAR and β-catenin. By taking into account data and observations obtained with different mouse models and employing different experimental approaches, it is shown that published data also contain substantial evidence that xenobiotic activators of CAR do not activate, or do even inhibit signaling through the β-catenin pathway. The review highlights new aspects of possible ways of interaction between the two signaling cascades and will help to stimulate scientific discussion about the crosstalk of β-catenin signaling and the nuclear receptor CAR.

## Signaling through β-catenin and its role in tumorigenesis

Numerous rodent studies have been published investigating molecular mechanisms of chemically induced liver carcinogenesis. About 20 years ago it has been shown that single treatment of mice with the genotoxic tumor initiator N-nitrosodiethylamine followed by chronic treatment with the non-genotoxic tumor-promoting compound phenobarbital, selects for the outgrowth of mouse liver adenoma bearing activating mutations in the *Ctnnb1* gene (Aydinlik et al. 2001). *Ctnnb1* encodes the multi-functional protein β-catenin. Comprehensive review papers containing detailed information about β-catenin, its functions and regulation have been published, for example see Behrens and Lustig (2004), Lustig and Behrens (2003), Shang et al. (2017), and Torre et al. (2011). In brief, two major functions of β-catenin are distinguished: on the one hand, β-catenin constitutes a part of the intracellular anchoring of cell–cell connections when associated with cadherin proteins. On the other hand, β-catenin can also function as a co-activator of transcription factors from the T-cell factor/lymphoid enhancer-binding factor (TCF/LEF) family in the so-called canonical Wnt/β-catenin signaling pathway. To this end, a cytosolic multi-protein complex tightly controls the levels of free cytosolic, non-membrane-bound β-catenin by catalyzing the phosphorylation of β-catenin at various amino acid residues near its N-terminus. Phosphorylation of these sites primes β-catenin for subsequent ubiquitinylation and proteasomal degradation. The activity of β-catenin as a transcriptional co-activator is physiologically controlled by so-called Wnt molecules, which bind extracellularly to Frizzled receptors. Activation of Frizzled receptors destabilizes the multi-protein complex responsible for β-catenin phosphorylation. This in turn leads to cytoplasmic accumulation and nuclear translocation of β-catenin. Nuclear β-catenin co-activates the transcription of TCF/LEF target genes, of which some, for example *MYC* and *CCND1*, are clearly linked to cell proliferation and survival, thereby providing a selective advantage to tumor cells that possess activated β-catenin signaling (Shang et al. 2017). In chemically induced mouse liver tumors, β-catenin activation was present due to activating mutations in exon 3 of the *Ctnnb1* gene, which eliminated phosphorylation sites essential for β-catenin degradation (Aydinlik et al. 2001). Overall, aberrant activation of β-catenin is one of the major drivers of tumorigenesis in various organs, both in humans and laboratory rodents, e.g. see the review by Shang et al. (2017). It should be noted that different ways of β-catenin activation prevail in individual tissues: for example, β-catenin activation in human colon carcinoma is frequently caused by deletions in *APC*, a gene encoding a protein that is part of the multi-protein complex regulating β-catenin phosphorylation and degradation; for review, e.g. see Raskov et al. (2014).

## Linkage of constitutive androstane receptor (CAR) and β-catenin

**Fig. 1:**
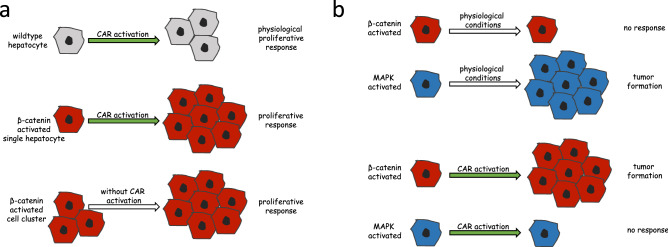
Schematic representation of the impact of CAR and/or β-catenin activation on the proliferation of hepatocytes and the formation of hepatocellular tumors. **a** CAR activation triggers a transient proliferative response in normal hepatocytes. Hepatocytes with activated β-catenin (due to gene mutations or transgene activation) do not proliferate under physiological conditions, but are stimulated to proliferate by the presence of CAR activators. **b** Under normal conditions, i.e. in the absence of a xenobiotic activator of CAR, pre-tumoral cells with activated β-catenin do not give rise to tumors, while other tumor precursor cell populations with activated MAPK signaling grow out to form hepatomas. Chronic treatment with a CAR activator inverts the situation and enables the selective outgrowth of tumors with activated β-catenin

In the abovementioned tumor initiation-promotion experiment, mouse liver adenoma with activated β-catenin had been exclusively detected after treatment with phenobarbital, but not in the absence of the tumor-promoting agent (Aydinlik et al. 2001). This is in contrast to tumor sub-populations bearing activating mutations in genes encoding key players of the mitogen-activated protein kinase (MAPK) signaling pathway (Aydinlik et al. 2001); see also schematic delineation in Fig. 1. The antiepileptic drug phenobarbital exerts its pharmacological activity by allosteric binding to the GABA_A_ receptor in neuronal cells (Czapinski et al. 2005). In the liver, the best-studied molecular target of the compound is the constitutive androstane receptor (CAR), which is indirectly activated by the drug (Elcombe et al. 2014; Whysner et al. 1996). The nuclear receptor CAR acts as a chemo-sensor for foreign compounds and induces the transcription of a battery of target genes related to drug and xenobiotic metabolism following exposure to CAR-activating compounds, while other CAR target genes link the receptor to changes in cellular energy metabolism and hepatocellular proliferation (Braeuning et al. 2011a; Elcombe et al. 2014; Honkakoski and Negishi 2000; Konno et al. 2008; Molnar et al. 2013). Recently, the epidermal growth factor EGFR has been postulated to represent a molecular target of phenobarbital in rodent liver (Mutoh et al. 2013).

The tumor-promoting effect of phenobarbital clearly depends on CAR activation, as proven by experiments with *Car* knockout mice (Yamamoto et al. 2004). Consequently, other CAR activators have also been shown to promote the growth of mouse liver tumors with β-catenin mutations, namely the rodent model CAR agonist TCPOBOP (Mattu et al. 2018), and the non-dioxin-like polychlorinated biphenyl PCB153 (Strathmann et al. 2006). The clear connection between phenobarbital-dependent tumor promotion and β-catenin is underlined by studies showing the lack of tumor promotion in mice with conditional hepatocyte-specific knockout of *Ctnnb1* (Awuah et al. 2012; Rignall et al. 2011). Inversely, β-catenin activation by other means than chemically-induced mutations of *Ctnnb1* also leads to β-catenin-activated cells that are promoted by phenobarbital. This has been demonstrated in an *Apc*-deficient mouse model, as well as in a study with transgenic mice where activating deletions of *Ctnnb1* in exon 3 were triggered by adenoviral infection with Cre recombinase (Braeuning et al. 2016; Dong et al. 2015).

CAR activation triggers a transient proliferative response of hepatocytes, thus inducing, together with hypertrophic effects, liver enlargement by means of a hyperplastic mechanism (Braeuning et al. 2011a; Elcombe et al. 2014). The amplitude of that proliferative response is also modulated by β-catenin. Conditional hepatocellular knockout of *Ctnnb1* substantially diminishes hepatocellular proliferation (Braeuning et al. 2011a; Ganzenberg et al. 2013), while the combined activation of CAR and β-catenin triggers an uncontrolled proliferative response of hepatocytes in mice (Dong et al. 2015). A very recent study links CAR activation-dependent transient hepatocyte proliferation to β-catenin signaling via activation of protein kinase B (AKT) (Yarushkin et al. 2020). Activation of β-catenin by *Apc* knockout in mouse liver triggers proliferation of β-catenin-activated hepatocytes also in the absence of CAR activation leading to severe hepatomegaly, if larger, connected clusters of these modified hepatocytes are present (Colnot et al. 2004), whereas phenobarbital treatment was necessary to stimulate proliferation of single *Apc*-knockout hepatocytes (Braeuning et al. 2016). CAR and β-catenin activation effects on hepatocellular proliferation are schematically summarized in Fig. 1.

Another relationship between β-catenin and phenobarbital/CAR activation has emerged from research aimed at understanding the molecular determinants of metabolic zonation in the liver, i.e. the differential regulation of gene expression in periportal and perivenous hepatocyte subpopulations. Here, it has been shown that the perivenous gene expression profile strongly depends on the activity of the β-catenin signaling pathway (Benhamouche et al. 2006; Berasain and Avila 2014; Braeuning and Schwarz 2010; Burke et al. 2009; Burke and Tosh 2006; Hailfinger et al. 2006; Sekine et al. 2006; Torre et al. 2011; Yang et al. 2014). Expression and induction of CAR target genes related to drug and xenobiotic metabolism preferentially occurs in the perivenous zone of the liver lobule (Buhler et al. 1992; Oinonen and Lindros 1998), related to the higher activity of β-catenin in these regions. More specifically, the drug metabolism-inducing cellular response to CAR-activating compounds is reduced in β-catenin-deficient hepatocytes (Braeuning et al. 2011a, 2009; Braeuning and Schwarz 2010; Ganzenberg et al. 2013).

Altogether, published literature indicates that (1) CAR activation in rodents selects for tumors with activated β-catenin, (2) both factors, CAR and β-catenin, are essential for this process, (3) CAR and β-catenin cooperate in the induction of proliferation and drug metabolism-related gene expression in non-transformed hepatocytes, thus suggesting synergistic effects of the activation of both pathways.

## Published hypotheses for molecular interactions of CAR and β-catenin

**Fig. 2:**
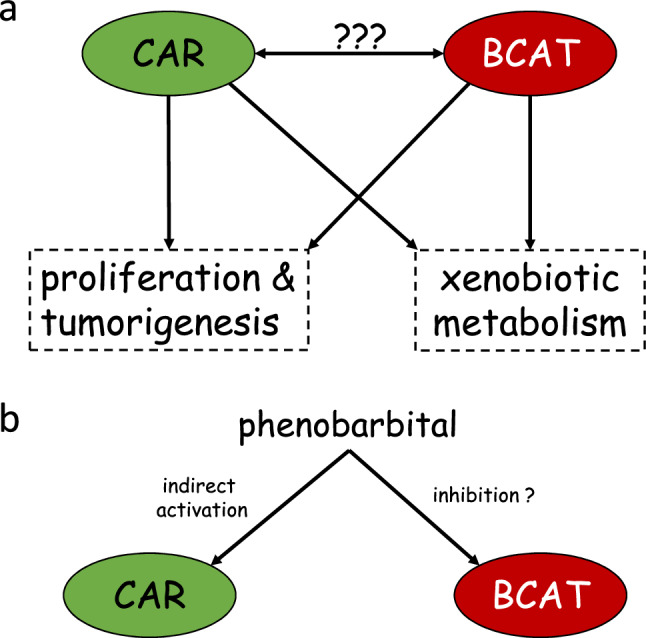
Scheme of interactions of CAR- and β-catenin-dependent signaling which may determine hepatocyte proliferation, tumor promotion and drug-metabolizing capacity. **a** Clear evidence is available for individual effects of CAR and β-catenin on the respective endpoints, as well as for a cooperation of the two pathways. There are still knowledge gaps with respect to the molecular details of the mutual interactions between the two players. **b** Phenobarbital as an indirect activator of CAR has been shown to exert CAR-independent effects on various molecular targets, including the β-catenin pathway. For more details, please refer to the main text

The exact molecular mechanisms by which both pathways, CAR and β-catenin, interact, is still not well understood (cp. Fig. 2). Very recently, an excellent review article about CAR-mediated liver cancer and its species differences has been published (Shizu and Yoshinari 2020). The authors of this article present an overview of different relevant signaling pathways and discuss species differences between rodents and humans, which are highly relevant for the assessment of rodent liver tumors induced by a CAR-dependent mechanism. In addition, a hypothesis for β-catenin-CAR interactions is presented, aimed to explain the effects observed with activators of the two signaling pathways. Based on data from published literature, the authors conclude that CAR might subsequently activate the β-catenin signaling pathway. Indirect evidence for this scenario comes from the fact that CAR and β-catenin share common target genes related to proliferation and drug metabolism (see above). For example, enhanced transcription of *MYC* is observed after CAR as well as after β-catenin activation (see above). Several drug-metabolizing enzymes are also under the control of both, CAR and β-catenin, as e.g. documented for some glutathione S-tranferase and cytochrome P450 enzymes (Giera et al. 2010; Schreiber et al. 2011). Moreover, Shizu and Yoshinari (2020) point towards a possible mechanistic connection between the two players via glypican 3. Overexpression of glypican 3 in mice suppresses hepatocyte proliferation and *Myc* gene expression after administration of model CAR activators (Lin et al. 2011). Glypican regulates β-catenin signaling by interacting with Wnt molecules (Kolluri and Ho 2019). It was thus concluded that possibly CAR activates β-catenin via a pathway involving glypican-3 (Shizu and Yoshinari 2020). An additional putative link between both pathways via p53/mouse double minute (MDM) 2 and p21 may exist (Shizu and Yoshinari 2020). The exact molecular nature of this interplay and its consequences for tumor cell survival and apoptosis, still need to be investigated in detail, as noted by the authors of the aforementioned paper. The hypothesis by Shizu and Yoshinari (2020) is corroborated by findings recently published by Yarushkin et al. (2020), showing an increase of β-catenin in mouse liver after single treatment with a CAR agonist.

Nonetheless, there are still major knowledge gaps regarding the molecular interactions of CAR and β-catenin signaling. This substantially complicates the interpretation of available data. Consequently, one might also come to a conclusion different to that of Shizu and Yoshinari (2020). Therefore, in the following review an alternative view of the data on the crosstalk between CAR and β-catenin interaction is presented. It is important to note that the goal of this exercise is not to disprove the hypotheses by Shizu and Yoshinari (which, in fact, would barely be possible based on the lack of specific molecular data of the interaction of the pathways). Instead, the aim is to highlight the ambiguity of some results and to stimulate further research in the field to clarify open questions.

## Stimulation of CAR by β-catenin?

The overlap of CAR and β-catenin target genes is considerable, especially with respect to cell proliferation and xenobiotic metabolism. This finding can be interpreted differently: activation of CAR might stimulate β-catenin-dependent signaling, β-catenin signaling might stimulate CAR-dependent transcription, and a more independent action at the gene promoters of common target genes is also possible.

The observation that *Car* is a target gene of the β-catenin pathway argues in favor of a regulation of the CAR pathway by β-catenin. Lowered levels of *Car* mRNA and protein have been observed in *Ctnnb1* knockout hepatocytes (Braeuning et al. 2009, 2011a), and elevated levels of *Car* mRNA are present in perivenous hepatocytes which physiologically possess activated β-catenin (Braeuning et al. 2006; Hailfinger et al. 2006). A TCF/β-catenin binding site is present in the upstream regulatory sequence of the *Car* gene (Gougelet et al. 2014). Moreover, *CAR* mRNA levels in human hepatocellular tumor tissue are higher if the tumor cells contain mutationally activated β-catenin (Gougelet et al. 2014). Nonetheless, an increase in CAR levels alone does not necessarily result in elevated functionality of the pathway, as this outcome depends also on other factors such as overall levels of the receptor, the availability of co-factors, and other parameters. For example, it has been shown that an increase in the cellular pool of another xeno-sensing receptor, the aryl hydrocarbon receptor (AHR), in mouse hepatoma cells does not result in elevated transcription of AHR target genes following stimulation of the cells with an AHR agonist (Schulthess et al. 2015).

For the AHR, a clear co-stimulatory role of β-catenin has been demonstrated. It involves interactions of adjacent transcription factor binding sites at the target gene promoter level (Schulthess et al. 2015), as well as physical interactions between the two proteins and enhanced activity of the AHR at its binding sites in the promoter regions of target genes in the presence of active β-catenin (Braeuning et al. 2011b; Schulthess et al. 2015). Similarly, β-catenin augments the function of hepatocyte nuclear factor (HNF) 1α at the *Cyp2e1* gene (Groll et al. 2016a). With respect to the findings with the aforementioned crosstalk of β-catenin with the AHR and HNF1α, two transcription factors with important roles in the regulation of hepatic gene expression and metabolism, it appears generally plausible that a similar way of interaction might occur between β-catenin and CAR. However, up to now there are no mechanistic data available to characterize this interaction. Nonetheless, it appears rather likely that the transcriptional activity of CAR in hepatic cells is positively affected by active β-catenin. This view would be also in line with the results from Dong and co-workers who showed synergistic effects between β-catenin and CAR activation at the level of proliferation- and cell survival-relevant gene expression (Dong et al. 2015).

## Inhibition of β-catenin by CAR?

As already detailed above, an activation of β-catenin by CAR has been proposed recently, based on a number of observations that link the two pathways (Shizu and Yoshinari 2020). Some other findings, however, may be interpreted in a way that they could also favor β-catenin inhibition by CAR, as will be detailed in the following chapters.

Metabolic zonation in the liver shows a clear preference for perivenous expression of numerous genes and proteins, as e.g. reviewed by Gebhardt (1992), Gebhardt and Hovhannisyan (2010), Kietzmann (2017) and Oinonen and Lindros 1998). The perivenous belt of hepatocytes expressing of high levels of many drug-metabolizing enzymes (including well-recognized CAR target genes) extends towards the periportal region following exposure to foreign compounds activating CAR or other chemo-sensors (Oinonen and Lindros 1998). This behavior, however, is not observed for all perivenous genes. Glutamine synthetase (*Glul*), a model target of β-catenin in the liver and physiologically expressed in a thin layer of hepatocytes around the central veins (Gebhardt et al. 2007; Loeppen et al. 2002; Werth et al. 2006), does not spread towards periportal regions after CAR activation (e.g. see Braeuning et al. (2009); Dong et al. (2015)). Even though *Glul* expression is regulated in a complex manner, it could be expected that a hypothetical, substantially increased activity of the β-catenin signaling pathway following activation of CAR by a respective xenobiotic agonist might lead to a broadening of the perivenous belt of *Glul*-expressing hepatocytes. It is important to note here that transgenic activation of β-catenin signaling alone in periportal hepatocytes is sufficient to induce a perivenous-like gene expression profile including high levels of the GS enzyme, irrespective of the position of a hepatocyte within the liver lobule (Schreiber et al. 2011). Instead, GS protein levels appear even to decline in perivenous hepatocytes of mice after administration of a model CAR agonist (Treindl et al. 2020), even though this finding might be biased by other factors affecting hepatocellular GS contents, such as CAR-induced hypertrophy and increased production of other proteins. Taken together, the abovementioned findings do at least not favor a scenario in which CAR activates β-catenin. Instead, also recent evidence at the mRNA level may suggest possible inhibitory effects of CAR activation on β-catenin signaling. It is interesting that the increase in *Axin2* mRNA, a direct β-catenin target gene routinely used to monitor β-catenin transcriptional activity, which is caused by transgenic activation of β-catenin in mice, is substantially lowered by simultaneous treatment with a model CAR activator (Dong et al. 2015).

The observation that tumors with activated β-catenin are promoted by the chronic presence of xenobiotic CAR activators does also not necessarily mean that CAR activation has a positive-regulatory effect on the strength of the β-catenin signal. Direct genetic activation of β-catenin signaling by point mutations or deletions in exon 3 of *Ctnnb1* affecting the important phosphorylation sites, as well as indirect activation via a loss of *Apc*, trigger very strong activity of the pathway by eliminating the most important physiological mechanism of regulation. Even though one cannot rule out that, in such a situation, small additional activation of β-catenin signaling can be reached in the presence of high concentrations of powerful CAR agonists, it appears unlikely that CAR activation exerts a very strong β-catenin-inducing effect in such a tumoral or pre-tumoral cell bearing mutational activation of the β-catenin pathway.

Strong activation of oncogenic signals is known to be able to induce cellular senescence and/or apoptosis, whereas more subtle or transient signaling is tolerated by the cells. This has been clearly document for RAS-dependent signaling (Serrano et al. 1997). Comparable to that, adenoviral activation of β-catenin in mouse hepatocytes has shown to induce senescence of infected cells (Dong et al. 2015). This corresponds with previous findings: Activation of β-catenin alone in a limited number of hepatocytes is not sufficient to trigger proliferation and outgrowth to hepatocellular tumors in mouse models of chemically induced liver carcinogenesis (Aydinlik et al. 2001; Mattu et al. 2018; Strathmann et al. 2006). Similarly, no pronounced proliferative response is observed in transgenic models of β-catenin activation or APC inactivation in a fraction of hepatocytes (Braeuning et al. 2016; Harada et al. 2002; Schreiber et al. 2011).

Senescence induced by β-catenin activation is suppressed by CAR activation (Dong et al. 2015). This has been shown using staining for senescence-associated β-galactosidase as well as mRNA and protein expression of relevant genes such as *Cdkn1a* (p21) (Dong et al. 2015). According to the hypothesis of β-catenin activation by CAR as put forward by Shizu and Yoshinari (2020), an even higher activity of the β-catenin pathway than already achieved by the activating mutation would possibly overcome cellular senescence. However, there is no direct experimental evidence available that CAR in fact activates β-catenin signaling under the conditions of the experiments performed by Dong et al. (2015). On the contrary, reduced mRNA levels of the model β-catenin target *Axin2*, as well as the reversion of β-catenin-dependent *Cdkn1a* induction by CAR activation (Dong et al. 2015) may point towards a reduction of β-catenin transcription activity. Such behavior would conform to a scenario where overwhelming β-catenin signaling induces senescence and this break is released by simultaneous activation of CAR. However, it remains to be elucidated whether CAR activation assists with releasing of the break by interference with β-catenin signaling per se, or by a more indirect, permissive action, e.g. by stimulating or inhibiting additional pathways which then impact on cellular processes also regulated in a β-catenin-dependent manner. There appear to be different ways how β-catenin-activated hepatocytes can be motivated to enter into massive proliferation. One way appears to be connected with the number of cells bearing the activated β-catenin. Single hepatocytes with activated β-catenin remain quiescent, whereas simultaneous activation in the majority of hepatocytes triggers a massive proliferative response (Colnot et al. 2004; Harada et al. 2002). This may suggest an active role of cell–cell-interactions in the regulation of β-catenin-dependent proliferation in the liver. As another way of inducing a proliferative advantage of β-catenin-activated cells, simultaneous transgenic activation of β-catenin and Ha-ras-dependent signaling has shown to substantially induce tumor growth in mouse liver not observed with β-catenin activation alone (Harada et al. 2004). Last, and most importantly in the present context, activation of CAR appears to be able to generate an environment where hepatocytes with activated β-catenin show proliferative behavior; e.g. see Braeuning et al. (2016) and Dong et al. (2015). Interestingly, CAR activation also favors the growth of normal hepatocytes with physiological β-catenin expression in an environment of surrounding *Ctnnb1* knockout hepatocytes (Braeuning et al. 2010; Rignall et al. 2011). It should also be noted that exposure to phenobarbital has been reported to induce mRNA expression of the Wnt signaling inhibitor *Wisp1* in mice (Luisier et al. 2014).

## CAR-independent effects of phenobarbital

A functional crosstalk between AMP-activated protein kinase (AMPK), CAR, the pregnane-X-receptor (PXR), hepatocyte nuclear factor (HNF) 4α and the peroxisome proliferator-activated receptor (PPAR) α in livers of mice exposed to phenobarbital has been described (Shindo et al. 2007; Tamasi et al. 2009). There is evidence for a connection between AMPK and β-catenin, suggesting a context-dependent activatory or inhibitory role of AMPK in the regulation of the Wnt/β-catenin signaling pathway (Park et al. 2019; Zhao et al. 2010, 2011). Phenobarbital has been shown to increase HNF4α mRNA levels and nuclear protein localization in a CAR- and PXR-independent manner (Bell and Michalopoulos 2006). Signaling through HNF4α and β-catenin are functionally linked via a double-negative feedback loop in epithelial-mesenchymal transition (Yang et al. 2013).

Many activators of CAR similarly activate the pregnane-X-receptor (PXR), another nuclear receptor with known relevance in the regulation of drug and energy metabolism. In a transcriptome-wide analysis of phenobarbital effects in *CAR*-knockout HepaRG cells it was found that only a fraction of genes regulated in wildtype cells was also induced or repressed in the *CAR*-knockout variant (Li et al. 2015). Some genes repressed in the HepaRG CAR-depleted cells after phenobarbital treatment appear to be associated with Wnt signaling suggesting a CAR-independent (potentially PXR-dependent) effect of phenobarbital on that pathway. It was shown that treatment with the model PXR ligand pregnenolone-16α-carbonitrile significantly augmented hepatocyte proliferation induced by CAR activation with CAR activators in wild-type mice but not in PXR-deficient mice (Shizu et al. 2013). These data suggest complex and synergistic effects of different nuclear receptors in hepatocyte proliferation. Not much, however, is known about a potential crosstalk between PXR and β-catenin. Available data suggest that PXR-dependent activation of xenobiotic metabolism-related gene expression is augmented by activated β-catenin in human HepaRG hepatoma cells (Thomas et al. 2015), while no pronounced effects of a *Ctnnb1* knockout on PXR-dependent target gene induction were visible in mouse liver (Braeuning et al. 2009).

In vitro, phenobarbital has been shown to inhibit β-catenin-dependent signaling in mouse hepatoma cells (Groll et al. 2016b). This occurs in a CAR-independent manner, because the cell line used did not express remarkable levels of CAR, and because a direct CAR activator did not exert comparable effects (Groll et al. 2016b). The relevance of this finding for the situation in vivo is not clear. Any conclusions would not be applicable to findings obtained with CAR activators other than phenobarbital; however, the antiepileptic drug has been in use as a model compound in many important studies. Assuming the theoretical possibility that phenobarbital indeed has an inhibitory effect on the β-catenin pathway in mouse liver cells in vivo, a scenario where CAR activation by phenobarbital causes subsequent β-catenin activation would appear unlikely. Instead, if phenobarbital activated β-catenin via CAR, the effect would probably be counteracted by CAR-independent β-catenin inhibition. However, as mentioned above, the in vivo relevance of this observation is unclear, making these considerations highly speculative. Nonetheless, the fact that β-catenin inhibition by phenobarbital is observed in vitro does not increase the likelihood of a scenario where β-catenin is activated via CAR, at least as long as phenobarbital is used as the CAR-activating foreign compound (cp. scheme in Fig. 2).

## Conclusion

In summary, the exact molecular details how CAR allows hepatocytes with activated β-catenin to proliferate, remains to be elucidated. The studies published so far contain data that may favor a stimulatory role of CAR towards β-catenin as suggested by Shizu and Yoshinari (2020), while an opposing view also appears justified with respect to the information available. Many aspects of the underlying molecular processes are still unclear, as is the contribution of the use of very different experimental models to the ambiguity of data. Future research will help to unravel the still unknown aspects of the complex interaction between CAR and β-catenin.
